# Molecular Responses of the Eukaryotic Cell Line INT407 on the Internalized *Campylobacter jejuni*—The Other Side of the Coin

**DOI:** 10.3390/pathogens13050386

**Published:** 2024-05-07

**Authors:** Anita Steinbach, József Kun, Péter Urbán, Tamás Palkovics, Beáta Polgár, György Schneider

**Affiliations:** 1 Department of Medical Microbiology and Immunology, Medical School, University of Pécs, 7624 Pécs, Hungary; anitani88@gmail.com (A.S.); palkovics.tamas@pte.hu (T.P.); polgar.beata@pte.hu (B.P.); 2 Hungarian Centre for Genomics and Bioinformatics, Szentágothai Research Centre, University of Pécs, 7624 Pécs, Hungary; kun.jozsef@pte.hu (J.K.); urban.peter@pte.hu (P.U.); 3 Department of Pharmacology and Pharmacotherapy, Medical School, University of Pécs, 7624 Pécs, Hungary

**Keywords:** *Campylobacter jejuni*, INT407 cell line, infection, WTA, apoptosis, tumorigenesis

## Abstract

*Campylobacter jejuni* is a zoonotic bacterium with the capacity to invade the epithelial cells during the pathogenic process. Several bacterial factors have been identified to contribute to this process, but our knowledge is still very limited about the response of the host. To reveal the major routes of this response, a whole-transcriptome analysis (WTA) was performed where gene expressions were compared between the 1st and the 3rd hours of internalization in INT407 epithelial cells. From the 41,769 human genes tested, altogether, 19,060 genes were shown through WTA to be influenced to different extents. The genes and regulation factors of transcription (296/1052; 28%), signal transduction (215/1052; 21%), apoptosis (153/1052; 15%), immune responses (97/1052; 9%), transmembrane transport (64/1052; 6%), cell–cell signaling (32/1052; 3%), cell–cell adhesions (29/1052; 3%), and carbohydrate metabolism (28/1052; 3%) were the most affected biological functions. A striking feature of the gene expression of this stage of the internalization process is the activation of both immune functions and apoptosis, which convincingly outlines that the invaded cell faces a choice between death and survival. The seemingly balanced status quo between the invader and the host is the result of a complex process that also affects genes known to be associated with postinfectious pathological conditions. The upregulation of TLR3 (3.79×) and CD36 (2.73×), two general tumor markers, and SERPINEB9 (11.37×), FNDC1 (7.58×), and TACR2 (8.84×), three factors of tumorigenesis, confirms the wider pathological significance of this bacterium.

## 1. Introduction

*Campylobacter jejuni* is one of the most important bacterial agents causing gastrointestinal infections with varying symptoms, ranging from mild to bloody diarrhea. The infection is common both in developed and under-developed countries. Outbreaks were reported in Eurasia, America, Africa, and even Oceania, which represents the global importance of campylobacteriosis. Epidemics caused by *C. jejuni* are not negligible, and these are registered more frequently in developing countries [1,2,3,4,5].

The typical source of infection is chicken meat, but the etiological agent can also be transmitted to humans through the consumption of contaminated water, milk, and meats. Additional means of infection are direct animal contact and inadequate hygienic–sanitary conditions in general [6,7].

Although there is a big difference among the isolates in their pathogenic potential, virulence, and genome organization, the general pathogenicity process of *C. jejuni* can be described with the following stages. *C. jejuni* enters the host intestine through the gastric acid barrier of the stomach and colonizes the mucosa covering the distal ileum and colon. During passage into the small intestine and the migration of the bacteria towards the mucus-filled crypts, *C. jejuni* reacts, presumably as an adaptive response, to the microenvironment of the current intestinal section, where it synthesizes a new set of proteins, facilitating their subsequent interaction with the host’s target cells. The flagella and the screw shape of the bacterial cells play an important role in reaching the epithelial cells through the mucus layer [8]. A group of adhesion proteins supports the binding of the bacterial cell to extracellular matrix proteins (ECMPs), including fibronectin and laminin. This process is facilitated by a whole arsenal of adhesion factors such as CadF, Peb1, Peb2, Peb3, Peb4, CapA, CjaA, FlpA, FbpA, JlpA, and DocA [9,10,11,12]. After adhesion, *C. jejuni* cells can penetrate enterocytes through the paracellular or transcellular routes. The role of HtrA in the pathogenic process was recently demonstrated, as this serine protease cleaves cell junctions and thereby opens the paracellular route of infection [13]. In contrast, Campylobacter invasion antigens (Cias) ensure the pathogen enters the cytosol of the host cell [14,15]. Through that process, the special role of flagella as a “molecule injector” was demonstrated [16].

As the first step of the invasion, the pathogen interacts with the host through biochemical signals, such as Campylobacter invasion antigens (Cias) [17,18]. As a result, a signaling cascade triggers the rearrangement of the host’s cytoskeleton, leading to the internalization of the bacterium in a vacuole [19]. At this point, a two-sided game begins in which the *C. jejuni* cell, in order to ensure its survival, tries to maintain the vacuolized form by avoiding its fusion with lysosomes. On the other side, the eukaryotic cell attempts to eliminate the invader. Some recent studies have outlined molecular changes, such as the expression of a capsule, lipo-oligosaccharide, different membrane transport systems, and also the activation of stress-related genes accompanying the invasion process and assuring the survival of the bacterial cell [9,20,21]. Other studies primarily focused on immunologic aspects such as the appearance of interleukins in the supernatant of invaded cells, showing increased expression of IL-8 due to infection [22] in a strain-dependent manner, as well as minor changes in the expressions of IL-1 and IL-4 in a time-dependent manner with a slight increase at the 1st and 4th hour and a subsequent decrease by the 24th hour following infection [23]. Other studies demonstrated the massive expression of TNF-alpha, suggesting that the immune response was shifted towards the Th-1 type [24].

One approach to understanding host–pathogen interactions and the interplay between bacteria and human cells is to perform a transcriptional analysis. The expressions of IL-4, IL-8, and IL-10 were recently investigated using RT-PCR in order to clarify the immunological aspects of *C. jejuni* infection and, more precisely, the potential roles of these ILs in the development of Guillain–Barré syndrome (GBS), one sequela of *C. jejuni* infection [25,26,27,28]. To the best of our knowledge, no analysis of other genes related to immune functions has been within the scope of transcriptomic-based methods.

Recently, whole-transcriptome analysis was used to outline those molecular changes that occur in the third hour of invasion in the *C. jejuni* cell [9]. Besides the marked upregulation of oxidative stress genes, outer surface structures, like adhesion-related factors, LOS, and capsule, were also upregulated, which strongly suggested the formation of a protective shield around the internalized bacterium cell. Using this strategy, the internalized bacterium cell seemingly separated itself from the harsh host environment. On the other hand, the internalized *C. jejuni* cell has actively interacted or combated with its new environment, which was represented by increased membrane and transport protein expressions, in that the membrane-integrated part of the flagellar apparatus also took part [9].

Nevertheless, our knowledge is still very limited concerning those changes that occur in the eukaryotic cell during the invasion and we do not have answers to such important questions such as through which routes the eukaryotic cell tries to eliminate or at least neutralize the invader and what cellular mechanisms become activated through the invaded eukaryotic cell that could be eliminated from the body as a potential source of further infection. These above issues are important aspects, as the outcomes of bacterial infections depend not only on the infectious agent but also on the host itself.

A medically important issue of *C. jejuni* infection is the development of certain late onset complications [29,30,31]. The most relevant of these are reactive arthritis (REA), Guillain–Barré syndrome (GBS), Miller Fisher syndrome (MFS), inflammatory bowel diseases, irritable bowel syndrome (IBS), and some forms of tumors [32,33]. Although we have some information concerning the involvement of certain serogroups and genotypes in these conditions [34,35], we do not have information about molecular changes preceding the formation of these sequelae.

The aim of this study was to gain insight into the molecular changes in the eukaryotic cell in the first stage of *C. jejuni* internalization. For this purpose, whole-transcriptome analyses (WTA) were performed from the 1st and 3rd hour of internalization and expression changes of groups of eukaryotic genes were compared. For the experiments, the INT407 eukaryotic cell line and the recently isolated and partially characterized, highly invasive *C. jejuni* strain CjTD-119 [9] was used.

## 2. Materials and Methods

### 2.1. Bacterial Strains and Growth Conditions

For this study, the *Campylobacter jejuni* CjTD-119 strain was used. This strain was partially characterized in a recent study [9], is a representative of a 190-piece strain collection, and is among those seven strains which were isolated from patients with bloody diarrhea. The strain, showing strong adhesion and invasion potential, was routinely grown on Charcoal Cefoperazone Deoxycholate Agar (CCDA) at 42 °C under microaerophilic conditions.

### 2.2. Preparing the INT 407 Cell Line for the Invasion Assay

The experiments were performed on a semi-confluent monolayer of INT407 human embryonic intestinal cell line (ATCC INT407 CCL-6) grown in 24-well culture plates for adherent cells (83.3922, Sarstedt, Germany). For monolayer formation, 3 × 10^5^ cells/plate (24 mL) were used and cultured in RPMI 1640 medium (BioWhittaker, Lonza, Switzerland) supplemented with 10% heat-inactivated (30 min for 56 °C) calf bovine serum (Sigma-Aldrich, St. Louis, MI, USA), 10,000 U/mL of penicillin, 10 μg/mL of streptomycin, and 0.5 mg/mL of neomycin, incubated overnight at 37 °C in a humidified, 5% CO_2_ incubator. After 24 h incubation, the cell monolayer was washed once with PBS, and then the bacterial cells were added as written below.

### 2.3. Infection of INT 407 with the C. jejuni Strain CjTD-119

For invasion, 12 h old cultures of the *C. jejuni* strain CjTD-119 were used. Bacteria were collected with a plastic loop from the blood agar plates and suspended in RPMI 1640. The optical densities of the suspensions were set to OD_600_ = 0.1, which equals nearly 10^8^ CFU/mL. A total of 100 μL from this suspension was added to each well containing the semi-confluent layer of INT407 human embryonic intestine (jejunum and ileum) cell line (MOI 100). Plates were centrifuged at 100× *g* for 10 min at room temperature to enhance the adhesion of the bacterial cells on to the surface of the eukaryotic cells and synchronize the invasion process. The plates were incubated at 37 °C with 5% CO_2_ for 1 h and 3 h, respectively.

Altogether, 3 plates (72 wells) were used for the experiments. Two plates were used for RNA isolations (1 h and 3 h), while the third was used to quantify the number of internalized *C. jejuni* cells at the first hour of invasion by performing the gentamicin protection assay (GPA). Briefly, after 1 h invasion, cells were washed three times with PBS and an additional 1h incubation was applied with 1 mL RPMI 1640 supplied with gentamicin (20 μg/mL, Aventis, Paris, France) to kill the attached but not internalized bacteria. After washing the plates three times with PBS, 1 mL Triton X-100 (0.1%) was added and, after thorough suspension, 10 μL volumes from the concentrated and the 100× diluted suspensions were spread-plated on CCDA medium. Plates were incubated at 42 °C under microaerophilic conditions and colonies were counted after 24 h.

### 2.4. Isolation of RNA from the INT 407

RNA isolations from the *C.-jejuni-*infected INT407 cells were performed in three parallels of the same timepoints (1st and 3rd hour of incubation). From one plate, 18 wells (3 × 6) were used for RNA isolations; therefore, one RNA sample contained the pool of six wells. Isolations from the INT407 cells were performed as described earlier [9], with some modifications. Briefly, before collecting, the cells wells were washed once with PBS. A total of 1 ml RNAprotect Bacteria Reagent (Qiagen, Hamburg, Germany) was applied to stabilize the RNA in each well. Following 5 min of incubation at room temperature, cells were trypsinized (Life Technologies, Carlsbad, CA, USA) and detached cells were collected with centrifugation (2000× *g* for 5 min). Collected cells were homogenized in RNAzol (Molecular Research Center, Cincinnati, OH, USA) in a 1.5 mL microcentrifuge tube. The tubes were frozen in liquid nitrogen (−196 °C), which was repeated three times in order to enhance the efficacy of RNA extraction. From that point, total RNA isolation was performed according to the instructions of the manufacturer. Briefly, 0.4 mL water was added to the homogenate and incubated for 10 min, followed by centrifugation (12,000× *g*, 15 min). Pelleted total RNA was washed twice with 0.4 mL 75% ethanol (8000× *g*, 2 min). Samples were solubilized in 100 μL deionized water. Total RNA concentration and purity was assessed with an ND-1000 Spectrophotometer (Nanodrop, Thermo Scientific, Carlsbad, CA, USA).

These isolation steps were performed on the relevant plates at the 1st and 3rd hour after infection. In each case, just before RNA isolation, CFU determination was performed from parallel wells at both the 1st and the 3rd hour of samplings.

### 2.5. Whole-Transcriptome Analysis

WTA was performed as described earlier [9]. Qualitative and quantitative measurement of the isolated RNA was carried out with Bioanalyzer (Agilent Technologies, Santa Clara, CA, USA) and Qubit (Life Technologies, Carlsbad, CA, USA). High-quality RNA samples were pooled for analysis. We used the SOLiD total RNA-Seq Kit (Life Technologies, Carlsbad, CA, USA) and RiboZero Prokaryotic rRNA Removal Kit (Epicentre, Madison, WI, USA). Remaining RNA was fragmented with RNase III, and the 50–200-nucleotide-long fragments were ligated to adaptors. Reverse transcription of these constructs was performed with ArrayScript RT kit (Thermo Fisher Scientific, Waltham, MA, USA). To purify the cDNA library, the Qiagen MinElute PCR Purification Kit (Qiagen, Hilden, Germany) was used and the library concentration was determined with SOLiD Library TaqMan Quantitation Kit (Life Technologies, Carlsbad, CA, USA). Emulsion PCR (ePCR) was carried out to amplify quality-controlled libraries on SOLiD P1 DNA Beads. Enrichment of template-positive beads was performed by hybridization with magnetic beads. Using terminal transferase and 3′ bead linker, template-enriched beads were extended at their 3′ ends. After placing the beads, containing clonally amplified DNA, onto sequencing slides, sequencing was carried out with the SOLiD5500XL platform (Thermo Fisher, Carlsbad, CA, USA), using the 50-base sequencing chemistry. Reads were aligned to the hg19 reference using BWA [36], and duplicates were marked and removed with Picard [37].

### 2.6. Reverse Transcription-Quantitative qPCR Validation

Levels of differentially expressed genes of RNA-Seq results were validated by randomly selecting six genes. They were chosen by the following criteria: 2 upregulated genes with a Fold Change (FC) >2 (coding for FNDC1 and ISL2); 2 genes with FC1 (coding for GAPDH and VMD1); and 2 downregulated genes with FC >2 (coding for MYOM1 and TMEM86A) (Table S1). For the analysis, the OriGene Human RT-PCR primer system was used (OriGene Technologies, Inc, Rockville, MA, USA) and the reactions were carried out with the suggested PCR conditions, such as activation: 50 °C for 2 min; pre-soaking: 95 °C for 10 min; denaturation: 95 °C for 15 s; annealing and elongation: 60 °C for 1 min. Melting curve analysis was performed between 95 °C and 60 °C.

### 2.7. Bioinformatics

The CLC Genomics Workbench 5.0.1 (CLC Biohttp://www.clcbio.com/ accessed on 16 January 2024, Qiagen, Hilden, Germany) was used for data analyses. Low-quality sequences, short sequences, and adapter sequences were removed in a trimming step by using Cutadapt [38]. Only sequences 45–50 nucleotides long were used for analysis. Sequences were mapped onto the human eukaryotic genome sequence Ensembl 66, using the TopHat pipeline v1.3.3 [39]. For each gene, expression levels were calculated as the average fragments per kilobase millions (FPKM) of all expressed exons. Transcript abundances were obtained using the MISO v0.4.1 tool [40]. Estimates were based on the existing transcript annotation (GENCODE v11). False positive hits were cured and manually removed. For further analysis, only those genes were considered that showed at least a 2.0-fold increase or decrease in their transcription levels. For heatmap analysis, normalized expression values of genes for the two samples were plotted on a heat map using the heatmap v1.0.12 package in the R language (v4.2.2). Genes with FDR *p*-value corrected Kal’s Z-test *p*-values < 0.001 were taken into account. The 50 most upregulated and 50 most downregulated genes, based on the fold change of normalized values, were selected for visualization.

### 2.8. Visualization of the Results

General distribution of the affected genes during the investigated time frame was visualized by volcano plot diagram, while visualization of functional enrichment results was performed by dot plot (or pathway enrichment bubble plot) diagrams and gene concept network diagrams (https://yulab-smu.top/biomedical-knowledge-mining-book/enrichplot.html, accessed on the 20 January 2024). Analyses were performed by using the freely available SRplot software (https://www.bioinformatics.com.cn), accessed on the 20 January 2024.

The volcano plot was created with the 2-based logarithm of the normalized fold change data and the −10-based logarithm of the *p* values. The X-axis was set between −5 and 5 and the y-axis between 0 and 6.

We examined the distribution of genes by naming biological or molecular functions; then, we looked at how many genes played a role in it and what their expression patterns were (upregulated or downregulated), after which we created a bar graph from the data.

For the enrichment analysis, we used the SRplot program (https://www.bioinformatics.com.cn/basic_local_go_pathway_enrichment_analysis_122_en), accessed on the 1 February 2024. As a first step, we selected the biological function of interest, then identified the affected genes with this biological function on the list and followed the instructions of the program.

## 3. Results

### 3.1. Comparison of Expression Changes of Genes Associated with Immune Functions

**Figure 1:**
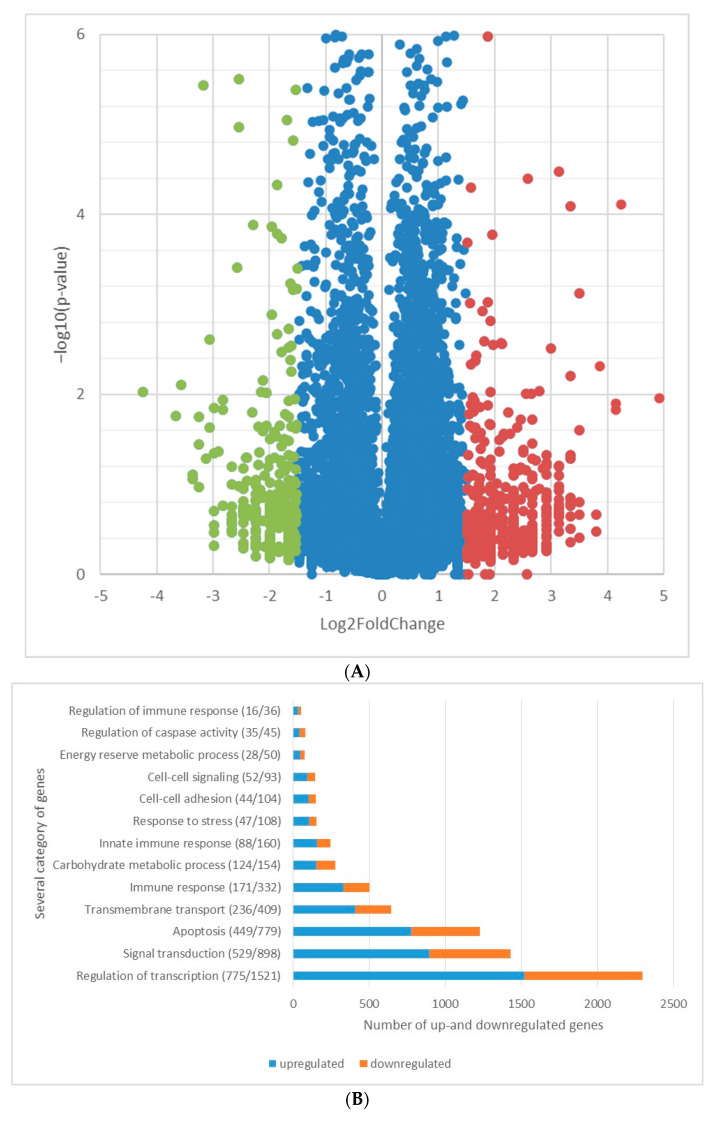

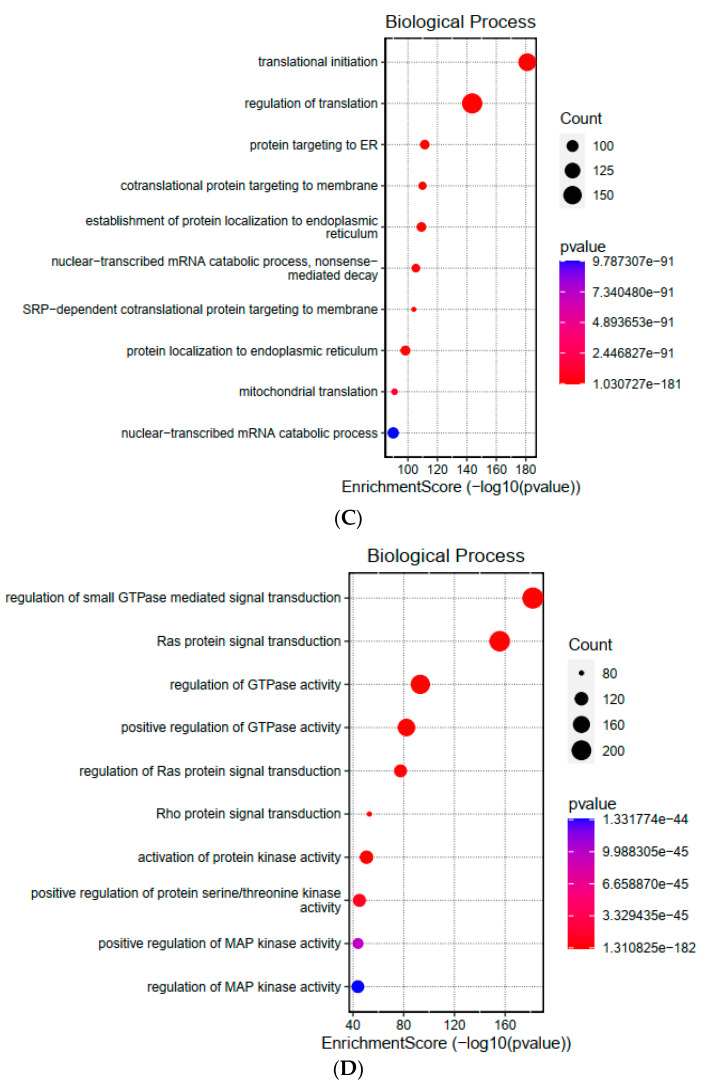

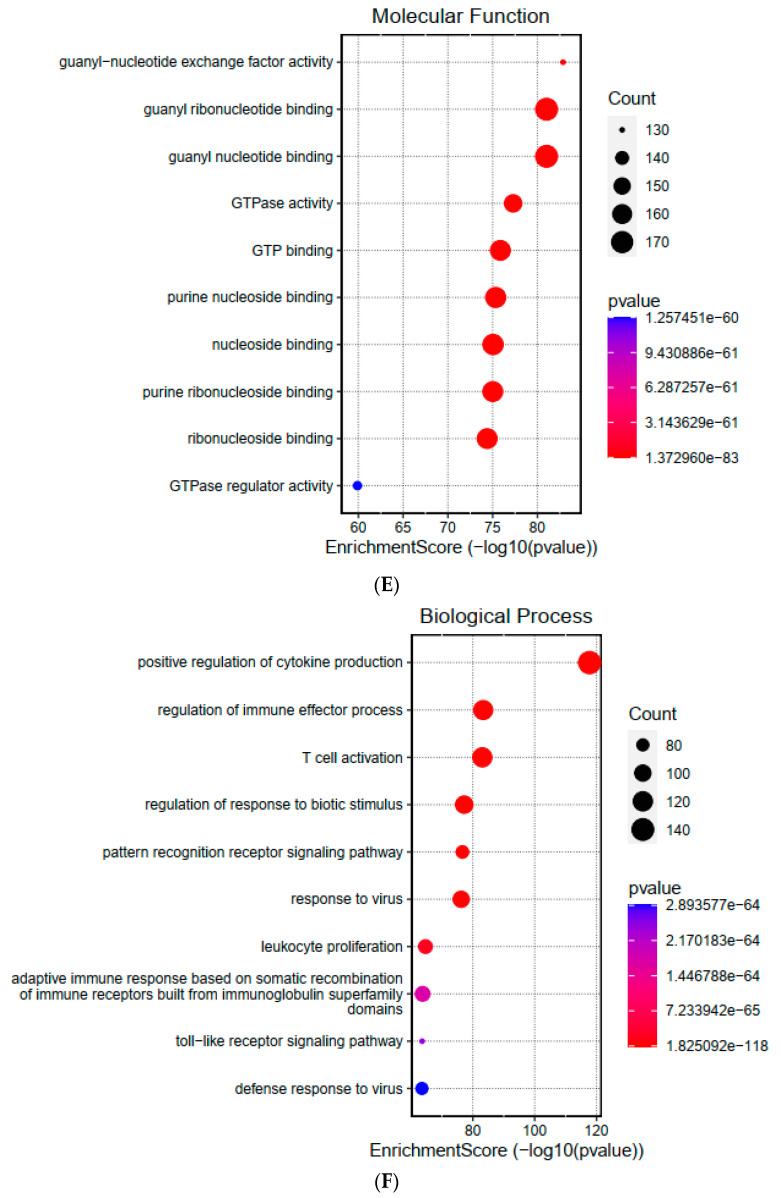

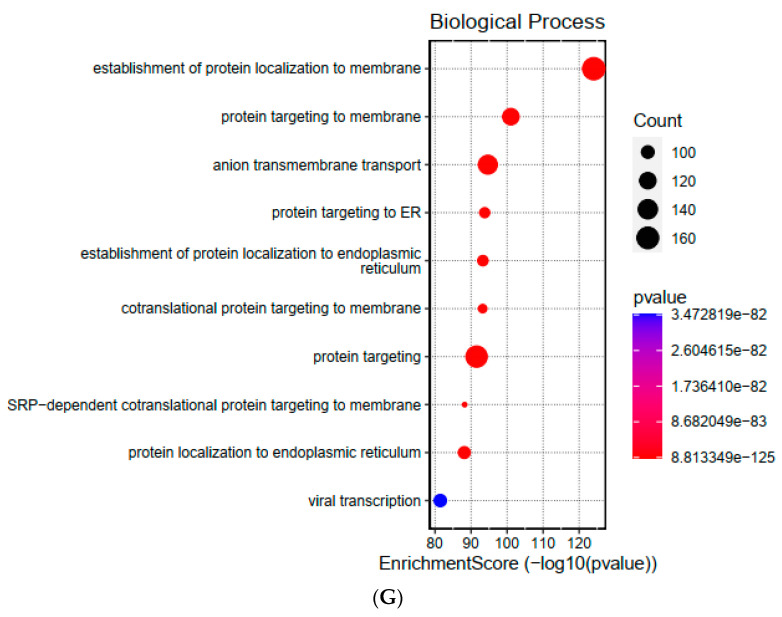
Distribution of the transcriptomic changes among the affected genes and functions of the eukaryotic cell line INT407 from the 1st and 3rd hour of the invasion with *C. jejuni*. (**A**) Volcano plot diagram shows the distribution of upregulated and downregulated genes of INT407. (**B**) Distribution of the upregulated and downregulated genes within categories of major biological functions. Distribution of affected functional gene groups within major biological categories such as (**C**)transcription and translation; (**D**) signal transduction; (**E**) apoptosis; (**F**) immune functions; (**G**) transmembrane transports were visualized by pathway bubble plots. (Diagrams were generated with the SRplot software: https://www.bioinformatics.com.cn) 12 March 2024.

The complete list of the 41,796 genes with their expression levels are listed in the Supplementary Table S2. Altogether, 19,060 genes and 22,734 pseudogenes and introns were identified by whole transcriptome analysis by mapping them onto the human genome. From among the active genes, 2764 were upregulated (fold change ˃ 2.0), while 2220 were downregulated (fold change ˃ 2.0) in the two hour time range between the 1st and 3rd hour after *C. jejuni* cells became internalized into the INT407 cell. Grouping of the genes with increased or decreased expression levels, based on their formerly confirmed or only hypothesized major physiological functions, is summarized in Figure 1.

Genes associated with immunological functions showing an altered expression are presented in Table 1.

Our data show that the infected human cells reacted to the *C. jejuni* infection with an upregulated immune response. Not only the genes responsible for innate immunity but also some others taking part in adaptive immune processes, were upregulated. The gene showing the highest expression change was *ULBP3* (ULBP3, Fc.: 10.10×), whose protein product, ULBP3, is related to MHC class I proteins and is an important regulatory protein of the natural immune system. Another gene with strongly upregulated expression was *CR1* (*CR1*; Fc.: 7.581×), coding for a transmembrane glycoprotein playing a part in various processes in the body, including adhesion and phagocytosis of immune cells [52], and which is also associated with several diseases [53]. A gene responsible for macrophage activation, *IFI44L*, showed a characteristic increase in expression (*IFI44L*, Fc.: 3.685×), which is among the highest within the group of genes affecting immune processes.

A slightly lower expression change (*CD74*, Fc.: 2.527×) was detected in the case of *CD74*, which performs many tasks in the eukaryotic cell. Its functions include (i) negative regulation of mature B cell apoptosis, (ii) positive regulation of neutrophil chemotaxis, (iii) positive regulation of T-helper type 2 immune response, and (iiii) negative regulation of apoptosis [46]. Additionally, it is a positive regulator of B cell proliferation, the extracellular signal-regulated kinases 1/2 (ERK1 and ERK2) and mitogen-activated protein kinases (MAPKs), both of them taking part in a diverse array of cellular processes [54].

Expression levels of four genes, *CD36*, *PROCR*, *CD209*, and *IL10RA* (*CD36*, Fc.: 2.73×, *PROCR*, Fc.: 2.527×; *CD209*, Fc.: 2.52×; *IL-10RA*, Fc.: 2.52×), all responsible for antigen processing and presentation, were also markedly increased. CD36 is an exogenous peptide and has a role in antigen presentation via MHC class I and positive regulation of the MAPKKK cascade. The function of CD209 (*CD209*, Fc.: 2.53×) is regulation of T-cell proliferation, antigen processing and presentation, and innate immune response [55]. IL-10RA is the cell surface receptor for the cytokine IL-10 [56].

IL-23R (IL-23 receptor, Fc.: 6.31×) is the cell surface receptor for the cytokine, which regulates immune cell activity. After binding to its receptor, IL-23 indirectly triggers a sequence of intracellular chemical signals, promoting inflammation to help build up a coordinated immune response against foreign invaders such as bacteria and viruses.

### 3.2. Affected Genes Related to Metabolic Functions

Among the metabolism-related genes, we found four whose expression was changed more than 2-fold over the study period. ABCD_2_ (*ABCD2*, Fc.: 11.37×) has a key role in the fatty acid homeostasis of peroxisomes, the oxidative organelles of eukaryotic cells [57]. TRPM6 (*TRPM6*, Fc.: 11.37×) is a selective magnesium channel [58], while ENPP3 (*ENPP3*, Fc.: 8.84×) is an ectoenzyme, which hydrolyses extracellular nucleotides, such as ATP, preventing ATP-induced apoptosis, and deregulates cytokine production.

Several other metabolism-related genes were found to be up- and downregulated (Table 2), with changes less than 2.0 in their expression level.

### 3.3. Comparison of Expression Changes of Genes Associated with Stress Responses

Concerning stress related genes, a drastic increase in the expression of *VNN1* (*VNN1*, Fc.: 379.08×) was detected (Table 3). This gene’s product contributes to tolerance to tissue damage and modulates the ability of the affected cell to cope with oxidative stress. Additionally, another stress gene, *LPO*, first described in connection with lipid peroxidation and strongly related to oxidative stress conditions, showed increased expression at the 3rd hour following infection (*LPO*, Fc.: 5.05×). Furthermore, a slight increase in the expression of three additional genes: *CHAC1*, *ADCYAP1R1*, and *RGCC* was detected (*CHAC1*, Fc.: 8.845×; *ADCYAP1R1*, Fc.: 3.791×; *RGCC*, Fc.: 3.791×). CHAC1, is thought to regulate the glutathione level and the oxidative balance in the cell. Lipid peroxidation controlled by LPO is a fundamental constituent of oxidative stress and free radical production [62], while RGCC has an effect on stress fiber formation [62]. The expression of *HSPA12B* (*HSPA12B*, Fc.: −2.374×), a heat shock protein [63], and *SCAMP5* (*SCAMP5*, Fc.: −3.165×), an inhibitor of endocytosis, was downregulated during the investigated time period [64].

### 3.4. Affected Genes Related to Apoptosis

At least 25 genes formerly hypothesized to have roles in the induction of apoptosis were also affected with more than 2-fold change in their expression levels (Table 4). Of these, 22 were found to positively affect the apoptotic process.

Concerning the positive regulators of apoptosis, the expression of *DCC* (*DCC*, Fc.: 5.68×), *DLC1* (*DLC1*, Fc.: 5.05×), and *CD27* (*CD27*, Fc.: 3.79×) has been documented in several different cells, while the expression of other genes, such as *FNDC1* (*FNDC1*, Fc.: 7.85×), until now has only been detected in specific cell types, such as cardiac cells. FNDC1 plays an important role in angiogenesis and is essential to hypoxia-triggered cardiomyocyte apoptosis [71]. However, recent studies have shown that aberrant expression of *FNDC1* is associated with tumorigenesis, for example, in gastric cancer [72].

Other affected genes are inhibitors of apoptosis. It has been reported that *SERPINB9* (*SERPINB9*, Fc.: 11.37×) protects cells from the immune-killing effect of granzymeB (GRB), released by lymphocytes [73].

**Table 4:** List of affected genes associated with apoptosis between the 1st and 3rd hour of internalization.

Genes	1 vs. 3	Function	Reference
*SERPINB9*	11.3726	negative regulation of apoptosis by inhibiting granzyme B	[74] Bird et al. 2014 [75] Kaiserman et al. 2010
*ACVR1C*	10.1089	regulation of apoptosis	[76] Asnaghi et al. 2019
*CHAC1*	8.8454	apoptosis in response to endoplasmic reticulum stress	[77] Zhou et al. 2023
*FNDC1*	7.8517	positive regulation of cardiac muscle cell apoptosis	[78] Das et al. 2017 [79] Yunwen et al. 2021
*G0S2*	6.9499	positive regulation of apoptosis	[80] Heckmann et al. 2013
*NFATC4*	6.31812	positive regulation of apoptosis	[81] Mognol et al. 2016
*HIC1*	5.8969	signal transduction resulting in induction of apoptosis	[82] Wang et al. 2017
*DCC*	5.6863	regulation of apoptosis	[83] Mehlen et al. 1998
*DLC1*	5.0545	induction of apoptosis	[84] Zhang et Li 2020 [85] Ullmannova et al. 2007
*CD27*	3.79087	induction of apoptosis	[86] Prasad et al. 1997
*CASP3*	2.7851	nuclear fragmentation during apoptosis	[87] Porter et Jänicke 1999

### 3.5. Genes involved in the Potential Development of Chronic Conditions

Expression changes of several genes previously hypothesized or verified to have potential roles in the evolvement of postinfectious pathological conditions was revealed in our study (Table 5). Four genes previously thought to influence the severity of Guillan–Barré syndrome (GBS) [88,89,90] were affected differently but moderately (PTGS2, Fc.: 1.188×; ANXA3, Fc.: 1.31×; CREB1, Fc.: 1.73×) by the 3rd hour of infection (Table 5). Characteristic upregulation of the genes *RELB*, *BIRC3*, and *NFKBIA* (*RELB*, Fc.: 1.625×; *BIRC3*, Fc.: 1.958×; *NFKBIA*, Fc.: −2.55×) associated with the progression of inflammatory reactions was detected. However, a higher expression rate was seen in the case of *ACE* (*ACE*, Fc.: 3.79×), a gene hypothesized to induce autoreactive TH1 and TH17 cells and suppress regulatory T cells and thus being involved in autoimmune responses of the body [91].

Another characteristic group of genes whose expression has changed over the time range studied and which have previously been shown to be associated with a pathological condition are genes linked to tumorigenesis. The *MUC4* and *MUC6* genes showed clear increases in their expressions, with FC values of 3.15× and 3.36×, respectively. A more drastic expression was detected in the case of three genes *SERPINB9*, *FNDC1*, and *TACRD2* (*SERPINB9*, Fc.: 11.37×; *FNDC1*, Fc.: 7.58×; *TACRD2*, Fc.: 8.84×). *SERPINB9* has been demonstrated to be significantly associated with the development of precancerous lesions [73]. In contrast, *FNDC1* promotes the invasiveness of gastric cancer and correlates with the appearance of peritoneal metastasis [72].

### 3.6. Markedly Affected Genes with Unknown Functions

As a result of this study, several genes with unknown function were revealed, whose expression was increased during the first stage of the internalization process of *C. jejuni* in the INT407 eukaryotic cell line. The six genes with the highest expressions are shown in Table 6. Several additional genes can be identified in the Supplementary Table S2.

### 3.7. Reverse Transcription-Quantitative qPCR Validation of DEGs

**Figure 2:**
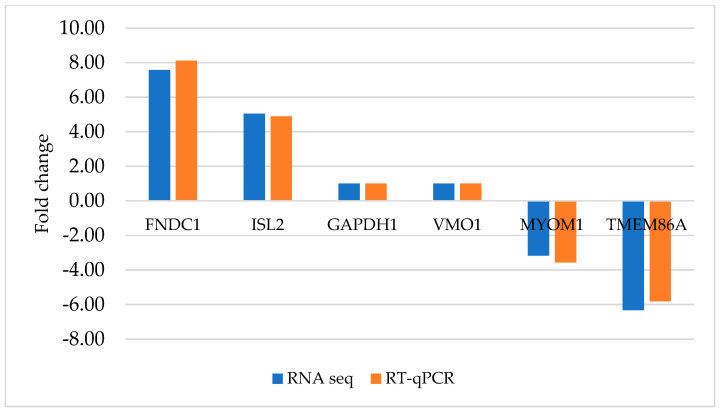
Validation of RNA-Seq results using RT-qPCR. Six genes, such as *FNDC1*, *ISL2*, *GAPDH1*, *VMO1*, *MYOM1*, and *TMEM86A*, were selected for validation. The mean CT values derived from three biological replicates of RT-qPCR are depicted by brown bars, while the RNA-Seq data are represented by blue bars.

The degree of gene expression of six randomly selected genes was confirmed with RT-qPCR. Comparing their changes in the gene expression detected by RNA sequencing and qPCR, we found high similarity suggested by a Pearson’s correlation coefficient of 0.93, confirming the accuracy and reliability of the RNA-Seq findings, as illustrated in Figure 2.

## 4. Discussion

Upregulation and downregulation of the 2764 and 2200 genes, respectively, from the 41,769 ORFs reflect intensive transcriptomic changes in the INT407 intestinal human cell line upon the invasion of *C. jejuni*. Our preliminary findings have revealed that one hour after infection, 85% of the *C. jejuni* cells became stably internalized. Our results provide insight into the background of this biological process in the short period of time between 1 and 3 hours after infection.

Activation of immune functions was represented by the increased expression of several relevant gene products. The prominent cytokine response, detected in the 3rd hour of infection and shown in the dot blot of Figure 1F, suggests that the invaded cell began to actively communicate with different immune functions. This is also demonstrated by the activation of T cells and Toll-like receptor signaling pathways. Since *C. jejuni* is an intracellular pathogen, the stimulation of defense responses related to viral infections was not entirely surprising (Figure 1F). Overexpression of *ULBP3* (ULBP3, Fc.: 10.109×) (Table 1), encoding for an MHC-I-related cell surface protein, is a good example since it demonstrates that the invaded cell prepares for antigen presentation, which is a crucial step in the immune recognition of cells infected with intracellular pathogens, like viruses or, in this case, with the invasive *C. jejuni* [42]. In addition, the activation of effector functions, especially those of T cells, is not surprising, since cytotoxic T cells are able to recognize infected eukaryotic cells [107], for which, however, proper processing of antigens is a prerequisite. This latter biological function was recently demonstrated to be supported by the gene products of *CD36* (*CD36*, Fc.: 2.74×), *PROCR* (*PROCR*, Fc.: 2.53×), *CD209* (*CD209*, Fc.: 2.523×), and *IL-10RA* (IL-10RA, Fc.: 2.53×) [48,49,50]. Antigen presentation on the surface of the affected eukaryotic cell cannot be effective without attracting immune cells. This is facilitated by the increased expression levels of CR1 (*CR1*, Fc.: 7.59×) and IFI44L (*IFI44L*, Fc.: 3.317×) [42,45], since both proteins make the infected cell more accessible to macrophages [52]. The previously mentioned cytokines and interleukins play an important role in the attraction of immune cells and the co-ordination of immunological processes, for which the expression of the proper receptors is inevitable. Increased expression rates of the IL-10 (*IL-10RA*, Fc.: 2.53×) and IL-23 (*IL-23R*, Fc.: 6.31×) (Table 1) receptors strongly suggest that these two interleukins contribute to the immune response induced by the vacuolized pathogen. IL-10RA activates the tyrosine phosphorylation of JAK1 and TYK2 kinases, two enzymes contributing to the alteration of the IFN-alpha/beta and gamma signaling pathways, thereby affecting the production of certain groups of cytokines [108]. The mild expression change of IL-4 (*IL-4I1*, Fc.: 1.25×) was in accordance with the findings of other authors [24], while the decreased expression of IL-8 (*IL-8*, Fc.: −2.74×) by the 3rd hour of infection is seemingly in contrast with an earlier finding [22]. A plausible explanation for this latter difference may be that our data were generated at the 3rd hour, while, in the study of Hickey, samples were taken 24 h following infection. Another possible reason may be the strain-dependent manner of the IL-8 expression rate [22]. In a recent study, the steep increase in the expression of the proinflammatory cytokines IL-6, IL-8, and IFN-γ and that of a regulator cytokine, IL-10, was detected at 5 h after infection [107]. Our results are in partial accordance with these findings, showing moderate increases in the case of IL-6 (IL-6R, Fc.: 1.7×), IL-10 (IL-10RA, Fc.: 2.52×), and IFN-γ (IFNGR2, Fc.: 1.4×), while the level of IL-8 transcript dropped (IL-8, Fc.: −2.74×) in the investigated time range (Table S2).

Based on our data, two possible outcomes can be outlined for the invaded eukaryotic cell. One option is survival by activating the immune system, while the other is to sacrifice the eukaryotic cell by apoptosis in order to eliminate the pathogen from the body [109]. In certain situations, on the level of the whole organism, it is much more rewarding to drive the infected cell towards apoptosis and localize the potentially emerging infection. This option is supported by the upregulation of those 10 specific genes, which are listed in Table 4, and by others shown in the dot plot graph of Figure 1E. It is still a mystery how the outcome will be decided but, based on the visual comparison of the Figure 1E,F, the activation of apoptotic genes appears to dominate. However, the expression of certain genes, such as *CD74* and *SERPINB9* (Table 1 and Table 4), two negative regulators of apoptosis, was increased. The gene product of *CD74* (Table 1) is also a positive regulator of the type-2 immune response [47]; thus, the increase in its expression turns the process towards the activation of the adaptive immune response.

Whether the result is immune system activation or apoptosis, both potential pathways drastically reprogram metabolism and thereby require the initiation and regulation of transcription and translation (Table S2). The dominance of these biological processes is observed from the data in the dot plot graph (Figure 1C), which is further strengthened by the co-ordinated increased expression of *ZNF491* (*ZNF491*, Fc.: 2.52×), *ZNF560* (*ZNF560*, Fc.: 2.52×), *ZNF516* (*ZNF516*, Fc.: 2.52×) [110], and *ESRRG* (*ESRRG*, Fc.: 2.52×) [111] and the enhanced expression of translation factors and other proteins involved in post-translational modification, such as GALNT5 (GALNT5 Fc.: 8.84×), MUC3A (MUC3A, Fc.: 6.31×) MUC6 (MUC6 Fc.: 3.36×), MUC4 (MUC4 Fc.: 3.15×) [112,113,114], PIWIL3 (PIWIL3 Fc.: 3.79×), PIWIL-4 (PIWIL-4, Fc:3.79×, F9 (F9, Fc.: 2.52×), and GFPT2 (GFPT2 Fc.: 2.52×). All these changes in the expressions of the above-mentioned genes represent a highly accelerated metabolic activity of the eukaryotic cell. Downregulation of *NDUFA13* (−4.11×), a negative regulator of translation [115], suggests that also the activity of genes or a group of genes controlled by this regulator is crucial in the battle between the invader and the host. During this encounter, it is essential to maintain the intracellular homeostasis, which is represented by the increased expression of TRPM6 contributing to Mg^2+^ homeostasis (Table 2), a key element of several enzymatic functions.

The markedly elevated levels of CHCA1 (*CHCA1*, Fc.: 8.84×) [66] and LPO (*LPO*, Fc.: 5.05×) [67] suggest that the eukaryotic cell, containing the vacuolized *C. jejuni*, undergoes a marked stress situation 1–3 h after infection. Decreased expression of CHRNE, a regulator of membrane potential (CHRNE, Fc.: −4.74×), assumes the development of osmotic shock. On the other hand, the drastically decreased expression of GAPDHS (GAPDHS, Fc.: −4.43×), an enzyme taking part in glycolysis [116], clearly indicates a partial slowdown of some parts of the metabolic machinery of the eukaryotic cell, which is a characteristic feature of stress conditions [111].

During stress situations, cells cease their nonvital functions and focus on energy saving. One feature of this may be the activation of the cell cycle arrest gene, *SESN2* (*SESN2*, Fc.: 3.23×) [111], thereby stopping cell proliferation, which is an energy-consuming process. Another example of energy savings is the decreased expression of PARVG (PARVG, Fc.: −3.95×) [112], a protein participating in matrix protein synthesis and matrix protein processing. The reduced expression of matrix proteins can either facilitate the killing of the infected cell by making it more accessible and, at the same time, more vulnerable to the damaging enzymes of macrophages. This hypothesis is supported by the powerful increase in EDN2 (EDN2, Fc.: 3.68×) [44], a macrophage chemoattractant, as well as the increased expression of the formerly mentioned ULBP3, which is responsible for natural killer cell activation.

The decreased expression of FAM132A, a negative regulator of inflammation [113], suggests an induced inflammation in the invaded INT407 cell. Activation of *RELB*, *BIRC3*, and *NFKBIA* [32] further supports this observation. It is important to note that, as a consequence of bacterial infections, inflammatory diseases and other pathological conditions, such as tumors, can develop. In this context, *C. jejuni* infection has been implicated in the development of Guillan–Barré and Miller Fisher syndromes (GBS and MFS). The reason for the more or less unaffected expression levels of the genes coding for PTGS2 (*PTGS2*, Fc.: 1.2×), ANXA3 (*ANXA3*, Fc.: 1.32×), and CERB1 (*CERB1*, 1.73×) (Table 5), three gene products considered to be associated with the development of GBS, may be that the effects of the affected genes manifest only after a longer exposition time or, as was recently suggested, GBS and, with it, the expression of these genes are associated only with certain *C. jejuni* serotypes [88,89,114,117]. Transcriptome analyses, following invasion experiments with the relevant serotypes, could help to clarify this issue.

The slight increase in the expression levels of genes associated with tumor genesis supports recent assumptions and findings that certain bacterial infections increase the risk of developing malignant tumors in the colon [118], the biliary tract [119], and the esophagus [120]. Although tumor genesis is a complex process about which our knowledge is still limited, the high expression levels of *SERPINB9* (*SERPINB9*, Fc.: 11.37×) and *TACR2* (*TACR2*, Fc.: 8.84×) [73,97], two proteins associated with tumor formation, supports the potential role of *C. jejuni* in the development of these pathological conditions.

## 5. Conclusions

The results of this study provide insight into a critical phase of *C. jejuni* invasion, in which the invaded eukaryotic cell faces severe stress situations and activates pathways steering the cell towards survival or death. However, our results provide only a brief insight into the molecular events that take place behind the scenes in the eukaryotic cell, yet they are groundbreaking because they allow for a genome-wide insight into how a eukaryotic cell tries to protect itself from an invader, at least in the first phase of the internalization process. Nevertheless, we have to keep in mind that our results originated from a fairly narrow timeslot of the probably otherwise stormy bilateral game between the intruder and the host. For this reason, follow-up studies have to concentrate on these aspects, either focusing only on certain selected factors or on complex sets of genes. Another issue is the parallel investigation of changes occurring in the pathogen and the host by WTA at several samplings timepoints of the infection, such as 0, 1, 3, 6, 12, 24, 48, and 168 h. Results of such a high-throughput study can not only confirm or cast doubt on the importance of certain genes for the success of the invasion process itself but also provide information about their potential roles in disease development. The potential factors identified could be targets for diagnostics or, through gene silencing, for therapy. If transcriptomic markers localize on the cell surface or are secreted into circulation or gut lumen, they can be potential targets for diagnostics. This could track the risk of developing inflammation, IBS, or tumors, which can contribute to early detection of diseases and immediate initiation of targeted therapy.

## Figures and Tables

**Table 1:** List of affected genes, associated with immune functions, between the 1st and 3rd hour of internalization.

Gene Name	Function	1 vs. 3 Experiment—Fold Change (Normalized Values)	Reference
*ULBP3*	regulation of immune response, natural killer cell activation	10.1090	[41] Sun 2003
*CR1*	innate immune response phagocytosis	7.581748	[42] Fällman et al. 1996
*IL-23R*	positive regulation of defense response to virus by host, inflammatory response	6.31	[43] Lupardus et Garcia 2008
*EDN2*	macrophage activation, signaling pathway	3.68557	[44] Grimshaw et al. 2002
*IFI44L*	defense response to virus, immune response	3.317	[45] DeDiego et al. 2019
*CD74*	negative regulation of mature B cell apoptosis, positive regulation of neutrophil chemotaxis, positive regulation of T-helper 2 type immune response, T cell selection, negative regulation of apoptosis, positive regulation of B cell proliferation, positive regulation of ERK1 and ERK2 cascade, antigen processing and presentation	2.5272	[46] Su et al. 2017 [47] Starlets et al. 2006
*CD36*	antigen processing and presentation of exogenous, peptide antigen via MHC class I, antigen processing and presentation of peptide antigen via MHC class I	2.73785	[48] Urban et al. 2001
*PROCR*	antigen processing and presentation, PROCR acted as a negative regulator of Th17 pathogenicity	2.5272	[49] Kishi et al. 2016
*CD209*	regulation of T cell proliferation, antigen processing and presentation, innate immune response	2.5272	[50] Preza et al. 2014
*IL-10RA*	inhibits the synthesis of proinflammatory cytokines	2.5272	[51] Liu et at. 1994

**Table 2:** List of affected genes associated with metabolic functions between the 1st and 3rd hour of internalization.

Gene Name	Function	Fold Change	Reference
*ABCD2*	very-long-chain fatty acid metabolic process	13.89	[57] Fourcade et al. 2009
*TRPM6*	Mg^2+^ channel, and uptake regulator	11.37	[59] van der Wijst et al. 2014
*ENPP3*	phosphate metabolic process, nucleoside triphosphate catabolic process	8.84537	[60] Tsai et al. 2015
*GFPT2*	glutamine metabolic process, fructose 6-phosphate metabolic process	2.52	[61] Wang et al. 2022

**Table 3:** The expression changes of genes associated with stress conditions.

Gene	Function	1 vs. 3	Reference
*VNN1*	response to oxidative stress, pantothenate metabolic process	379.08	[65] Zhang et al. 2017
*CHAC1*	apoptosis in response to endoplasmic reticulum stress	8.84537	[66] Mungrue et al. 2009
*LPO*	response to oxidative stress	5.05	[67] Kovács et al. 1996
*ADCYAP1R1*	multicellular organismal response to stress	3.791	[68] Ressler et al. 2011
*RGCC*	positive regulation of stress fiber formation, cell cycle regulation	3.791	[69] Wang et al. 2011
*HSPA12B*	response to stress	−2.374122	[70] Zouein et al. 2013
*SCAMP5*	response to endoplasmic reticulum stress	−3.165496	[64] Noh et al. 2009

**Table 5:** List of affected genes associated with the development of chronic conditions between the 1st and 3rd hour of internalization.

	Gene Name	1 vs. 3	Reference
Guillan–Barré s. disease severity (GBS)	*PTGS2*	1.188331	[90] Chang et al. (2012)
	*ANXA3*	1.315609	[89] Hughes et al. (1978)
	*CREB1*	1.732517	[89] Hughes et al. (1978)
Inflammatory	*RELB*	1.624660	[92] Breuer et al. 2013
	*BIRC3*	1.958618	[92] Breuer et al. 2013
	*NFKBIA*	−2.553647	[92] Breuer et al. 2013
Autoimmune inflammation	*ACE*	3.79087	[93] Connell et al. 2012
General cancer markers	*TLR3*	3.79087	[94] Wang et al. 2015
	*CD36*	2.73785	[95] Wang et Li 2019
Tumorigenesis	*SERPINB9*	11.37262	[73] Wang et al. 2021
	*FNDC1*	7.581749	[72] Jiang et al. 2020
	*TACR2*	8.845373	[96] Yu et al. 2012 [97] Jianfeng et al. 2021
Gastric cancer	*GALNT5*	8.84537	[72] Jiang et al.2020 [98] Guo et al.2018
	*MUC6*	3.36	[99] Marín et al. 2012
Pancreatic cancer	*KRAS*	2.011985	[100] Chang et al. 2020
	*SMAD4*	1.34157	[101] Xia et al. 2015
	*BRCA2*	1.231316	[102] Naderi et Couch. 2002
	*NBL1*	5.054499	[103] Olakowski et al. 2009
	*MUC4*	3.15	[104] Singh et al. 2007
Oxidative stress in the intestine	*VNN1*	379.087	[105] Pinho et al. 2022 [106] Kang et al. 2016

**Table 6:** List of the six genes with unknown functions, showing the highest expression values, which were identified from the internalization stage of *C. jejuni* in the INT407 eukaryotic cell line.

Gene Name	Fold Change
ST20-MTHFS	18.95437
*PRR4* (NW_003571047 44554..48182)	11.3726
*C12ORF55*	10.109
*NDUFA3(NW_003571054 77272..81394)*	10.109
*COL11A2* (NT_167245 4411775..4441552)	7.581748
*ADCK5* (NT_037704 165142..185869)	3.79087

## Data Availability

All data are freely available in the manuscript and in the Supplementary material. There are no restrictions with the usage of these published data.

## Supplementary Materials

The following supporting information can be downloaded at: https://www.mdpi.com/article/10.3390/pathogens13050386/s1, Table S1: Whole transcriptome data of the eukaryotic genes in the 3rd hour of internalization with *C. jejuni* strain CjTD-119; Table S2: Primers, used for RT-PCR validation.
