# Supplementary material for: Molecular Responses of the Eukaryotic Cell Line INT407 on the Internalized Campylobacter jejuni—The Other Side of the Coin
Source: Pathogens. 2024 May 7;13(5):386. doi: 10.3390/pathogens13050386 (PMC11124400; doi:10.3390/pathogens13050386)
Supplement: Supplementary file 1 [file pathogens-13-00386-s001.zip › Supplementary Table S2 Primers.pdf]

Supplementary Table S2

Selected candidate gene primer characteristics. Six genes were selected randomly from the higher-, middle- and lower hits for RT-PCR validation.

**Gene**	**Forward**	**Reverse**	**Fold Change**	***p*-Value**
FNDC1	TCATCTTGGGATGCGCTACCA	GGCAGAAGTAGTGTCTCCAGGA	7.58175	0.45148
ISL2	AGAGACGGGAAGACCTACTGCA	AGCACTCGATGTGGTACACGCT	5.05449	0.29145
GAPDH	GTCTCCTCTGACTTCAACAGCG	ACCACCCTGTTGCTGTAGCCAA	1	1
VMO1	GCTTTAATAGCGGAGGTGGGA	TTGGCTCCTTTTCTGTTCTGT	1	0.043363
MYOM1	TGTCAAGGCTGTCAGTGAGGAG	GAAGCATTCGCTTACTGCGGAG	−3.16549	0.54961
TMEM86A	CTCATCTGGCAGGACCAAGGAT	CATCACCAGACCTGTCCGAAGA	−6.33099	0.31581
